# Book Reviews

**Published:** 1808-09

**Authors:** 


### CRITICAL ANALYSIS

OF THE

### RECENT PUBLICATIONS

ON THE

### DIFFERENT BRANCHES OF PHYSIC, SURGERY, AND MEDICAL PHILOSOPHY.

*Description and Treatment of Cutaneous Diseases. Order III. Rashes. Part II. On the Urticaria, Roseola, Purpura, and Erythema: and Order IV. Bullæ. On Erysipelas Pemphygus and Pompholyx.* By ROBERT WILLAN, M. D. F. A. S. Physician Extraordinary to the Fever Institution, and to the Public Dispensary in London.

THIS number completes the first Volume of Dr. Willan's valuable work. Having so often given our opinion of the merits of the author, we shall proceed at once to offer our analysis of this part of his labours.

The divisions, or as the author calls them, varieties of Urticaria, are six in number, all of which he includes under Linnæus's general description of *Sudamina* pruriginosa, inæqualia, ruberrima, dilatabilia, fugacia, recidivantia, furfuraceo-evanescentia: Hectica brevis, benigna.

It will readily appear that all the varieties cannot be applicable to this definition. The word *Sudamina* is neither classical nor correct. It might do very well for Linnæus, who, finding it impossible to attend to etymology, always defined as he proceeded. But Dr. Willan's own description is much more to the purpose, and we believe, unobjectionable in every future part of his arrangement.

"The Urticaria, or nettle-rash, says he, is characterized by round, oval, or longitudinal elevations of the cuticle, usually denominated wheals, (def. ix.) which have a white top surrounded with diffuse redness. Some of the orbicular wheals resemble tubercles in form, (def. viii.) but they are not permanent, nor have they any tendency to suppurate. The nettle-rash, though on many occasions accompanied with fever, is not contagious."

This character is as clear as words can well make it. But in referring to the *Definitions*, we find (def. ix.) a vesicle called *Bullæ*, also; which, in the present number, is confined to the fourth order. This relates, however, more to the external form than the genuine character, which is very properly remarked by the author. We have, therefore, only made this slight observation, to show that drawings cannot always give a satisfactory resemblance of such appearances.

The six varieties comprehend, Urticaria febrilis; U. evanida; U. pertans; U. conferta; U. subcutanea; U. tuberosa.

Urticaria febrilis being the most general, is first in order. It is, says says Dr. W. " always attended with considerable disorder of the constitution. The symptoms preceding the eruption, are, pain and sickness at the stomach, head-ach, great languor or faintness, with a disposition to sleep, a sense of anxiety, an increased quickness of the pulse, and a white fur on the tongue. In two days, or sometimes later, after these symptoms, the wheals appear with an efflorescence in patches of a vivid red, or sometimes nearly of a crimson colour. They are preceded by fits of coldness and shivering, and are attended with a most troublesome itching or tingling, which is greatly aggravated during the night, and which prevents rest for many hours. In order to avoid this inconvenience, I have known many persons sleep on a sofa without putting off their cloaths, as their distress begins immediately on uncovering the body. The patches often coalesce so as to produce a continuous redness: they appear on most parts of the surface, but they are diffused particularly on the shoulders, loins, nates, thighs, and about the knees. They extend likewise to the face; and there is sometimes a red circle round the palm of the hand, accompanied with a sensation of violent heat. They appear and disappear irregularly, first on one part, then on another, and they may be excited on any part of the skin by strong friction or scratching. During the day, the efflorescence fades, and the wheals in general subside, but both of them return with a slight febrile paroxysm in the evening. The red patches of efflorescence are often elevated above the level of the adjoining cuticle, and form dense tumours, with a hard distinct border: the interstices are of a dull white colour. When the patches are numerous, the face, or the limb chiefly covered with them, appears tense and considerably enlarged. At the latter end of the disorder, the eyelids are red and tumefied, and there is often a swelling and inflammation on the sides of the feet. On the appearance of the eruption, the pain and sickness at stomach are in general relieved, but when it disappears, those symptoms return. The whole duration of the febrile nettle-rash is seven or eight days. As the eruption declines, the tongue becomes clear, the pulse returns to its usual state, and all internal disorder ceases: the efflorescence exhibits a light purple or pink colour, and then gradually disappears, being succeeded by slight exfoliations of the cuticle."

The complaint, though rarely dangerous, is yet extremely troublesome, from the perpetual itching and uneasiness it creates. On some occasion the sickness and languor, which preceded the eruption, has produced fainting. Dr. Willan relates a case in which it proved fatal. The subject was a man about 50 years of age, and of intemperate habits. The complaint occurred early in August, beginning with nausea and great pain in the stomach, encreased by pressure, thirst, quick pulse, and slight delirium at night. On the 3d and 4th days, the skin was covered with nettle-rash, and the stomach relieved. On the 5th day the rash disappeared, and the other symptoms returned with more violence than at at first. On the 6th, though the eruption re-appeared on the face, yet the violent constitutional symptoms continued; and in the evening the patient died.

We should, in this instance, consider the rash as one of the symptoms, and not as the cause of all this violent constitutional affection. It is but justice to our author to admit, that by his quotations, he seems aware of this himself. From Sennertus it appears, that he sometimes considers this rash as preceding bilious fevers, and threatening the worst form of that complaint. Dr. Cleghorn remarks the same of those violent tertians which were so destructive in the Island of Minorca.

Urticaria febrilis, it is very justly remarked, occurs oftener in summer than winter, and still more frequently in those who indulge their appetite too freely: is frequently a symptom of teething, attended with its usual fretfulness, a white tongue and quick pulse. In children from two to ten it is also frequent; and among adults, men are more frequently affected than women.

Some very useful practical facts are next related, on the effect which certain substances taken into the stomach produce on the skin, and frequently on the whole constitution. These are principally almonds, mushrooms, herrings, crab-fish, muscles, and lobsters. The whole of this passage is interesting, and two important enquiries arise from it. First, is there any thing poisonous in these substances, or in any of them, viz. muscles and mushrooms? or is there only a susceptibility in certain constitutions to receive such impressions? In muscles there seems every reason to believe that the latter is really the case. As to mushrooms, when we consider their great variety, it is to be regretted, that botanists have not yet informed us with some certainty, which species has been found deleterious, or whether any one has been uniformly so in every constitution.

The second Inquiry is suggested by Dr. Willan from the conclusions of "some physicians, That Urticaria generally originates from indigestion, or some poisonous substance taken into the stomach, even though, in a few cases, it may seem to have been produced by a sudden check to perspiration, without any previous internal disorder." This we think sufficiently answered by two remarks of the author; namely, that the disease is sometimes connected with teething in children; and that it does not often return in the same person. Dr. Withering's opinion, that the rash in scarlatina, arises from the secretion of the glands of the fauces being conveyed into the stomach, and thence acting as a poison, &c. might have been spared, as well as the solitary case which follows, when offered in favour of Dr. Withering's opinion.—In the mode of treating Urticaria, we could not expect much novelty. A caution, however, is given against antimonial preparations, which sometimes operate with such violence, as to occasion fainting. We are by no means convinced that any vomits whatever are necessary.

The following is our author's description of Urticaria evanida.—

 "Without

"Without fever, and without any extensive efflorescence on the skin. The eruption consists sometimes of round wheals (Pl. xxv. Fig. 1.) at other times of longitudinal elevations of the skin (W), resembling those which are produced by the stroke of a whip. They are all white at the top, but in some there is a slight redness at the base. Though often hard and elevated, they do not contain a fluid, or tend to suppuration. They are occasionally seen on every part of the body, but they are most numerous on parts which are closely covered. The eruption appears and disappears many times in the course of a day and night, according to the temperature of the air, or to the exercise used by the person affected. It may be excited on any part of the body, in a few seconds, by strong friction or scratching, but the wheals presently subside again. A violent itching, mixed with a sensation of tingling or stinging, attends the eruption. These sensations are most troublesome to patients while they undress, and for some time after they have lain down in bed, when the eruption is usually most vivid.

"The disease thus continues, with repeated eruptions, more or less extensive, for several months, or even for years. It occurs most frequently in persons of the sanguine temperament, especially in those who have a delicate stomach and an irritable skin. The occasional causes are heat, fatigue, watching, agitation of mind, or anxiety, and a too stimulating diet.

"I have observed that females are much more liable to this complaint than males. Dr Heberden, however, remarks, 'Males and females are equally liable to the nettle-rash: and I have observed it in all ages from childhood to decripid old age. Constitutions tainted with strumous or harrassed by rheumatic and hysteric complaints, or broken down with intemperance, palsies, and age, have all been, as far as I could judge, equally fitted for the disorder; but not more so than the soundest state of health in the vigour of life, to which all other complaints were unknown.'

"The last observation is, I think, incorrect. Persons affected with the Urticaria evanida may be capable of business and occasional exertion, but they are often low-spirited, and they seldom remain for twenty-four hours without some bodily uneasiness, as headache, lassitude, pain and sickness at the stomach, aching in the limbs, or an oppressive languor."

As this very irksome disease rarely yields to remedies, and is often produced by very slightly irritating substances applied to the skin, or by certain articles of food, Dr. Willan very properly directs the attention of the practitioner to a minute regard to these organs: yet candidly acknowledges, that in some cases, a total change of diet has produced no permanent effect on the disease: occasional laxatives he has found most serviceable of all internal remedies, and warm sea-bathing as an application to the skin.

In Urticaria perstans, the wheals are stationary, though the surrounding efflorescence is more transient. Heat excited from any cause, cause, produces itching: the disease is, for the most part, confined to the upper parts of the body, and continues for about three weeks. The stomach is often disordered before the eruption appears. The disease is sometimes shortened by moderate doses of aq. kali puri.

Urticaria subcutanea occurs at distant periods, and continues only a few days at each return; but the itching and tingling of the skin continue almost without intermission. The complaint in the beginning is confined to one spot, but becomes afterwards more extensive. Persons affected with it are liable to diseases in the stomach, and cramps in the lower extremities, and seldom have any sensible perspirations.

In the last division, Urticaria tuberosa, "the wheals increase to a large size, forming hard tuberosities, which seem to extend deeply into the muscular flesh, and occasion a contraction in the sinews, with total inability of motion, and a sensation of pain in the bones. These tumours are usually whitish at their tops: they rise on the arms, thighs, loins, and calf of the leg, and are very hot and painful for several hours. The eruption, in all cases under my observation, took place at night, and before morning it wholly disappeared, leaving the patient weak, languid, and sore, as if he had been bruised, or had undergone much fatigue.

"The Urticaria tuberosa often proves tedious and obstinate; I have known it continue, as above described, upwards of two years, with but a few short intervals. The only causes to which it could with probability be attributed, in the instances presented to me, were irregularities in diet, violent exercise taken by persons usually sedentary, and the too free use of spirituous liquors."

"I have, continues our author, adopted Dr. Frank's denomination of this complaint, and will subjoin one of his cases, which, however, differs in some particulars from the above statement. Adolescens viginti annorum, crapulis non parùm deditus, a prandio corripitur nauseâ et vertigine tenebricosâ. Intumescit mox, rubroque colore suffunditur facies; tumores verò palmæ latitudinem habentes, et colore rubro sed obscuro instructi, cum pruritu ad animi deliquium usque intolerabili, universam corporis, sed femorum in primis, superficiem occupare cernuntur. Accedit horror, ac demùm rigor, extremitates potissimùm inferiores violenter concutiens, sub quo exanthemata in altas, et superficiem longè ac latè superantes, seu informes offas, increscunt. Pulsus nunc duri et pleni observantur, mortisque ægrotantem consternit formido. Ad noctem, subsequitur calor, sub quo vix formati ad cutem tumores imminuuntur, ac tandèm, reliquo tamen ad vultum rubore ac universali pruritu, penitùs evanescunt. Ast verò eorundem mox locum exanthema asperrimum, digitosque sub attactu vix non offendens, miliaribus ferè simile, sed nigro ad apicem puncto insignitum ac pellucidum, subintrat. Hoc facto, imminuitur anxietas, orisque summa comparet amarities. Die morbi secundâ, purgante alvus medicinâ laxatur, quâ occasione, ægroto lectum cum frigi-

 diore diore aurâ commutante, posterius hoc exanthema iterùm disparet, ac primi denuò ad cutem revertuntur tumores. Alterna hæc exanthematis efflorescentia eodem per sex dies tenore continuatur, quo tempore, sectâ adinterim venâ, et antiphlogisticis et evacuantibus ægrotanti porrectis remediis, ægritudo disparuit omnis."

Some remarks follow, from the Greek and Arabian writers, which evince the learning and industry of the author, but contain very little information for the reader. With these, and a quotation from Ingrassia, the third division of the third ORDER concludes.

The fourth division of this order, comprehends Roseola. "By this title, says Dr. Willan, I mean to express a rose-coloured efflorescence, variously figured, without wheals or papulæ, and not contagious. The Roseola* can scarcely be considered as idiopathic, but though connected with different fevers, and, perhaps, with different states of the constitution, it requires notice in an arrangement of cutaneous appearances. I will, therefore, describe its principal varieties under the denominations Roseola æstiva, Roseola autumnalis, Roseola annulata, Roseola infantilis, Roseola variolosa, Roseola vaccina, Roseola miliaris."

The note is a very necessary appendage, and we recommend every reader, in the perusal of these remarks, and still more of the work, to avoid confounding the terms used by the author, with those of former writers. We enforce this the more, because we have already had occasion to remark, that when Dr. Willan adds the definition of other authors, he is not always sufficiently careful in keeping the attention of the reader to the precise meaning to which he confines his own terms.

The Roseola estiva is sometimes preceded by feverish symptoms for a few days. The eruption on the first day may be easily mistaken for the measles, and even for scarlatina, and it is on that account necessary to attend to every part of its future progress. The patches are usually larger than the measles, and die off by becoming only of a deeper roseate-colour, instead of the desquamation which follows the other disease.

Roseola autumnalis is so trifling a complaint, that nothing, we conceive, but the author's wish to render his work complete, would have induced him to notice it.

Roseola annulata is distinguished in its appearance by the annular form it assumes. The rings are at first small, but gradually dilate. The disease is extremely irksome, from the irritation it excites on the skin, and frequently continues for two, three, or four months: but a case is subjoined, in which it seems to have lasted more

"* I use this term arbitrarily. A few writers have employed it instead of Rossalia, (page 289,) which denotes the Scarlatina. The Roseola, mentioned by Severinus, Lib. VII. De reconditâ abcessorum naturâ, is left indefinite. Quæst. V.

more than two years, nor are we acquainted with the issue at this time.

Roseola infantilis relates properly to nothing more than those red patches, or splashes, which, during dentition, so often appear in different parts of the skin, where it is exposed to the open air. Dr. Willan has noticed a few other appearances, which he seems to have introduced on this occasion, because they were not easily referable to any other division.

The Roseola variolosa is nothing more than the general efflorescence which often precedes the small-pox eruption, whether inoculated or casual. The former is accurately described by Dimsdale; the latter by many writers on the small-pox. A very useful paragraph closes this division; in which the author remarks, that the scattered efflorescence which attends the eruption of inoculated small-pox, and which sometimes appears in distinct casual small-pox, has often been considered as a coincidence of small-pox and measles.

Roseola vaccina appears about the same time as variolosa, but is much less frequent.

Roseola miliaris often attending a miliary eruption.—"In Typhus, or contagious malignant fever, observes our author, an efflorescence also takes place occasionally, resembling, in its distribution, the specimen of Roseola, exhibited Pl. xxvi. Fig. 1.—but of a darker hue. I observed such a rash on the 15th day, in one case of fever, which terminated on the 17th day. In other cases, it precedes the formation of purple spots and vibices, and, in others, it is seen early in the disease, but remains only for a short time, without any material consequences."

This concludes the author's fourth division of the third order.—We have been very short in our remarks and extracts, because almost the whole appears to us applicable to the various diseases which this kind of eruption usually precedes; and, in our opinion, the author would have done better to have considered the few instances in which the disease is idiopathic, by themselves, and afterwards to have taken those, in which it is symptomatic in a single division, marking the slight differences between each, by opposing them to each other. By making separate paragraphs, and even divisions, he has only burthened the reader's memory, and charged it with distinctions which cannot always be marked, and are never important. This peculiarity in the actions of the articular vessels, has been marked by every writer who has described accurately any cutaneous eruption. The author has referred to several writers on the inoculated and casual small-pox, as well as on vaccination: we shall just remark two others on a different disease, who have spoken of the efflorescence as a frequent forerunner of many cutaneous eruptions.

Mr. Hunter says, "The appearances upon the skin generally show themselves in every part of the body; first in discolourations, making the skin appear mottled; many of them disappearing, while

 others others continue and increase with the disease." Treat. on Ven. Dis. P. 319.

This description might be uncertain, were it not for the following note attached to it.—" This is not peculiar to this disease; it often takes place in small-pox."

Dr. Adams, in his account of secondary venereal symptoms remarks. " In a few days the skin becomes generally of a mottled red. This is not, indeed, always the case; and when it is so, this appearance is not the real disease, but an affection of the skin, which often precedes small-pox, and other eruptions." Morbid Poisons, Page 165.

We have given these references, not to show any disrespect to the author, but to point out a mode in which he might have saved himself some trouble, and his readers still more: for we cannot suspect in Dr. W. any intention to assume the air of more profound investigation, by making unnecessary distinctions.

The fifth division of this order, is *Purpura*, by which term the author " proposes, with Riverius and some others, to express an efflorescence, consisting of small, distinct, purple specks and patches, attended with general debility, but not always with fever. Three striking varieties of the complaint, thus defined, may be denominated Purpura simplex, Purpura hæmorrhagica, and Purpura urticans, no one of which is contagious".—All these, particularly the second, have so near an affinity with stomacace, or sea-scurvy, that if they are really different, we wish the author had accurately marked the distinction. It is unnecessary for us to add, that sea-scurvy is now no longer imputed to the mere salt in salted provisions, but to " a sedentary mode of life, impure air, anxiety of mind, want of proper food." We should have been more cautious in this remark, on so judicious a writer, did not the nature of our work afford him an opportunity of giving us, and a large portion of his readers, that information which we shall be happy to receive from such a source.

The author remarks, that in the treatment of this disease, he should recommend moderate exercise in the open air, a generous diet, and the free use of wine, Peruvian bark, and vitriolic acid. If our suggestions are right, might it not be worth the trial to substitute the fresh vegetable acid for the vitriolic or marine. We had written thus far, when, in re-perusing the article, we met with the following passage. " Cases of Purpura seem to have been studiously multiplied in periodical publications, and in medical and chirurgical miscellanies. I consider it under all the forms described, as pertaining to scurvy, though it is not always attended with sponginess of gums and discharge of blood from them, according to the definition of scorbutus in nosology. Whether my readers agree with me in this opinion or not, they will, I think, allow, that a general view of the symptoms of sea-scurvy or land-scurvy cannot possibly form a part of the present work."

This judicious paragraph is not entirely satisfactory to us. It might might have done from an ordinary author; but Dr. Willan should have begun his account, by telling us that he could see no difference between Purpura and the scurvy of different authors. Land-scurvy is also an objectionable term. We know of no disease by that name; and if the author means Scorbutus, or Stomacace, as it appears by land, he should have expressed himself in that manner.—We trust our remarks will not be thought severe, or to intend any thing more than what we observed in our review of the former number, viz. that Dr. Willan has lived long, and worked hard enough to give the result of his own remarks, without encumbering himself with the opinions of others, any further than as they may illustrate his meaning.

The remainder of this chapter is devoted to petechial spots in the fevers usually called Typhus.

The fourth division of this order is Erythema; as the definition of Roseola did not admit papulæ, or tubercles, it was necessary to make this division. But on other accounts, we wish they could have been included in one, or could have succeeded each other, so that the recollection of the first, might have assisted the reader in contrasting it with the last. As it is, we shall content ourselves with transcribing the author's definition, taking notice of one of his varieties.—"The Erythema, says he, is a continuous redness of some portion of the skin, attended with disorder of the constitution, but not contagious. This appearance, though in general symptomatic, merits our attention, as it should be carefully distinguished from other eruptions, particularly from the contagious exanthemata." Instead of admitting among the varieties any effects produced by violence, as some have done, the author proposes to limit the subject under the following divisions. Erythema fugax; E. læve; E. marginatum; E. papulatum; E. tuberculatum; and E. nodosum. We shall only notice the tuberculatum; not on account of any observations we can offer, but that the reader may be aware of a disease which happily does not often occur, but which, on that account, may be less suspected.

"Erythema tuberculatum, exhibits irregular patches of red efflorescence, diffused, at first, round tumours slightly elevated, and of the size represented Pl. .1 Fig. 9. The small tumours are not so hard as tubercles (Def. VIII.): they are not attended with pain or uneasiness, nor do they seem disposed to suppurate. They subside in seven or eight days, but the patches of Erythema continue a week longer, then turn livid, and gradually disappear.—This eruption extends over the arms, neck, shoulders, thighs, and legs. It commences with fever, sometimes in paroxysms as severe as those of a quotidian ague. In the intervals of the paroxysms, the patient is affected with great languor and faintness. During the second week of the eruption, the fever gives place to a state of weakness, morbid irritability, and restlessness, which is sometimes succeeded by hectical symptoms."

Dr. Willan afterwards adds, that this disease yields to none of those remedies which have proved successful in the other varieties' and that the only three cases in which he saw it, terminated fatally.

With this division concludes the order of Rashes. The fourth order, Bullæ, shall be noticed in our next.

---

*Reports of the Royal Colleges of Physicians, of London, Dublin, and Edinburgh, on Vaccination; with Introductory Remarks, and other Papers.* By JOSEPH ADAMS, M. D. F. L. S. Physician to the Small-Pox and Inoculation Hospitals, and to the New Finsbury or Central Dispensary. Printed for the Benefit of the Small-Pox Hospital, 8vo. 1808.

THESE Reports have already appeared in our own, and most other Journals. The introductory remarks contain little besides a statement of the dreadful ravages of the uninoculated small-pox; the inconveniences attending inoculation; and the advantages of Cow-pox, as well as its security.

The additional papers consist of one, shewing the near resemblance of the mildest small-pox to the cow-pox, illustrated by some well selected cases, and a succession of peculiarly mild inoculations from the same source; a second, contains several cases in the observation of the author and others, in which cow-pox was attended with secondary eruptions and many feverish symptoms, so as to produce a considerable resemblance to small-pox: the third, a correspondence between Dr. Hervey, Register of the College of Physicians, and the Author, by which it appears, according to the opinion of the latter, that the progress of the cow-pox has been more retarded by the indiscreet zeal of its friends, than by the violence of its enemies.

---

*A Letter on Vaccination, or the Propriety of Inoculating Infants for the Cow-pox, considered; Addressed to those whose Example may Influence the inferior Orders.* By T. W. WADLEY, Calne, 8vo.

WE cannot expect any thing very new on this hacknied subject; and we regret to see the Author insist so much on the necessity that vaccination should be performed by a member of the college of surgeons. In our opinion, the "good woman who gains her livelihood by initiating the infantile pupils in the noble science of letters," with a few practical instructions, is as fit to vaccinate as the member of any college, and some advantages might attend such an operator. She would be more likely to see the whole progress of the inoculation by the daily attendance of her scholars; she ought, however, to be obliged to keep an exact register of all her patients, which she might as easily do, as of her account current with their parents.

---

*Vaccination Vindicated from some Prevailing Errors.* By JAMES RUMSEY, of Amersham, Bucks. 8vo. London.

THE learned Author has chosen for his motto, *Veritas prevalebit.* We are precisely of the same opinion; and think, by this time, if if an author has nothing very new to offer, the question may be left to the decision of the public, who will ultimately form their own opinion. At the same time, we have no wish to discourage writings which may be serviceable in removing local doubts, but they can be no longer interesting to our readers.

