# Erratum

**Published:** 1808-10

**Authors:** 


*Erratum.* P. 279, line 10, from the bottom, for *articular* read *cuticular.*

